# Morphological Study of Bio-Based Polymers in the Consolidation of Waterlogged Wooden Objects

**DOI:** 10.3390/ma15020681

**Published:** 2022-01-17

**Authors:** Zarah Walsh-Korb, Ingrid Stelzner, Juliana dos Santos Gabriel, Gerhard Eggert, Luc Avérous

**Affiliations:** 1 BioTeam/ICPEES-ECPM, UMR CNRS 7515, Université de Strasbourg, 25 rue Becquerel, CEDEX 2, 67087 Strasbourg, France; jugabriel.contato@gmail.com; 2 Staatliche Akademie der Bildenden Künste Stuttgart, Am Weißenhof 1, 70191 Stuttgart, Germany; ingrid.stelzner@rgzm.de (I.S.); gerhard.eggert@abk-stuttgart.de (G.E.); 3 Landesamt für Denkmalpflege im Regierungspräsidium Stuttgart, Berliner Straße 12, 73728 Esslingen am Neckar, Germany; 4 Department of Physical Chemistry, University of São Paolo, IQSC-USP, São Carlos 13560-970, Brazil

**Keywords:** freeze-drying, waterlogged wood, natural polymers, structure, archaeology, conservation

## Abstract

The removal of water from archaeological wooden objects for display or storage is of great importance to their long-term conservation. Any mechanical instability caused during drying can induce warping or cracking of the wood cells, leading to irreparable damage of the object. Drying of an object is commonly carried out in one of three ways: (i) air-drying with controlled temperature and relative humidity, (ii) drying-out of a non-aqueous solvent or (iii) freeze-drying. Recently, there has been great interest in the replacement of the standard, but limited, polyethylene glycol with biopolymers for wood conservation; however, their behaviour and action within the wood is not completely understood. Three polysaccharides—low-molar-mass (M*_w_*) chitosan (M*_w_* ca. 60,000 g/mol), medium-molar-mass alginate (M*_w_* ca. 100,000 g/mol) and cellulose nanocrystals (CNCs)-are investigated in relation to their drying behaviour. The method of drying reveals a significant difference in the morphology of these biopolymers both ex situ and within the wood cells. Here, the effect these differences in structuration have on the coating of the wood cells and the biological and thermal stability of the wood are examined, as well as the role of the environment in the formation of specific structures. The role these factors play in the selection of appropriate consolidants and drying methods for the conservation of waterlogged archaeological wooden objects is also investigated. The results show that both alginate and chitosan are promising wood consolidants from a structural perspective and both improve the thermal stability of the lignin component of archaeological wood. However, further modification would be necessary to improve the biocidal activity of alginate before it could be introduced into wooden objects. CNCs did not prove to be sufficiently suitable for wood conservation as a result of the analyses performed here.

## 1. Introduction

A large number of artefacts of cultural importance are organic in nature [1], of which wood is a particularly common, but highly important, material [2]. The degradation processes of many of these artefacts have been influenced by waterlogged environments, for example, lacustrine pile dwellings, means of transportation, tools or war and trading ships, such as the 16th century warship *Mary Rose*, the 17th century warship *Vasa* and the Etruscan harbour ships [3,4,5,6,7,8]. The waterlogged, mainly anoxic environments, from which they are excavated, do a remarkable job in preserving these objects for many centuries [9]. Upon excavation, however, problems begin to appear. Apart from mechanical damage, the combination of an oxygenated atmosphere and water within the wood provide an ideal environment for the rapid proliferation of bacteria and fungi, as well as chemical modes of degradation [10,11,12,13]. Drying of the object is, therefore, extremely important for long-term preservation efforts; however, dimensional instability during drying is often a major concern. When bound water is removed from the cell wall, shrinkage of the wood can occur. The development of contractile capillary forces during natural air-drying can lead to collapse of the degraded wooden structure [14]; thus, methods such as solvent-drying, freeze-drying and supercritical drying have been investigated [15,16,17,18,19,20]. To avoid shrinkage of the wood and strengthen the degraded object, free water is often exchanged for an aqueous polymer solution, typically poly (ethylene glycol) (PEG). The higher molar mass and much lower vapour pressure of PEG compared to water allows it to remain in the wood cells during drying [21]. In recent years, much work has been carried out examining the long-term behaviour of this common wood consolidant [15,22]. Mortensen et al. [21] observed that the natural aging of PEG produces formic acid, which exacerbates degradation of the acid-sensitive cellulose and holocellulose within the wood. Almkvist and Persson found that the formation of acidic by-products was accelerated by the presence of Fe^3+^ ions in the wood from degraded iron bolts and fastenings [23,24]. Moreover, mechanical instability during the period of display can also be problematic [25,26,27]. Bardet et al. [28,29,30] and Bjurhager et al. [4] observed that the use of high concentrations of hygroscopic low-molar-mass PEG consolidants could, over time, lead to plasticisation and deformation of wood cells [31].

These discoveries resulted in great interest in the development of alternative consolidants, mainly bio-based and biopolymer systems [26,27,32,33,34,35,36,37]. According to IUPAC, biopolymers can be defined as polymers directly extracted from biomass, such as proteins or polysaccharides [38]. While bio-based polymers have been extracted from natural resources, they have been modified, resulting in a composition that is not directly found in nature. Ultimately, the use of bio-based polymers and biopolymers is seen as a way to limit the use of fossil-based polymers with potentially damaging degradation from by-products. For simplicity, the term biopolymer will be used throughout the text, although chitosan, the deacetylated form of chitin, is technically a bio-based polymer rather than a biopolymer.

Recently, links have also been drawn between the overall weight of the consolidant within the wood and its long-term mechanical stability, indicating that mechanical failures due to excessive strain on weakened timber increase with increasing consolidant weight [25]. Although the molar mass of many potential biopolymer treatments is significantly higher than PEG, the amount of biopolymer needed to produce the same mechanical response is often dramatically lower. Walsh et al. showed that similar rheological properties can be obtained from solutions of biopolymers at 0.5–1 wt.% as well as for PEG solutions of approx. 50 wt.% [27]. Thus, overall reduced consolidant weight is a particular advantage of biopolymer treatments. However, the behaviour of these biopolymers in situ, their structure upon drying, an important factor for evaluating future re-treatability [35], and their biological resistance, a requirement for their applicability [15], were not examined.

Research has also shown that the efficient removal of free water from wood, and the method by which it is removed, also contribute to long-term stability. Freeze-drying has become a key technique for the gentle and controlled removal of water, as the wood is maintained in a frozen state while water is sublimed from the structure, avoiding cell wall collapse. The combination of freeze-drying with a consolidant that replaces water and bulks the remaining wood cells, which acts as a cryoprotector during freezing but does not sublime, further reduces damage. The majority of studies on drying behaviour in the presence of consolidants have focused on PEG and the effect of the freeze-drying conditions only [39,40,41,42,43,44,45]. However, conservation of waterlogged wooden and non-wooden objects with the bio-based polymer Klucel (hydroxypropylcellulose, M*_w_* = 80,000 g/mol) has been reported and has recently shown its applicability and suitability, as well as that of a range of other alternative polymers, to the freeze-drying process [46,47,48]. Concerns about the susceptibility of cellulose-based consolidants to the same degradation issues as the wood itself may, however, be an issue for their widespread application [27,32]. Moreover, the high molar masses of these biopolymers could retard their diffusion into the wood, which has been observed in the treatment of beech spatulas with Klucel EF [48] and archaeological oak with chitosan [34]. Experiences from the conservation of wooden objects reveal many examples where only the hull is consolidated but not the core, resulting in fragility [49].

As mentioned previously, solution concentrations of biopolymers are often significantly lower than PEG solutions, which can overcome issues of viscosity during treatment, as seen in Table 1 (experimental section), but leads to concerns over the density of the treatment within the wood.

However, in biomass, organisation tends to play the major role in the mechanical properties with many examples of strong and mechanically adaptable systems, where organisation within the structure contributes to exquisite mechanical properties, e.g., nacre, bone, teeth and wood itself [50]. Recent literature has also highlighted the usefulness of controlled drying techniques in the preparation of polysaccharide-based aerogels designed to mimic the high mechanical strength of biomaterials [51]. Thus, it is supposed that the structure of the polymer after drying, in addition to its molar mass, would affect the mechanical properties of the treated wood, much in the way structuration of proteins and biopolymers dictate the strength and stability of many aspects of biomass. The structure of the consolidant can also play a major role in the long-term preservation of archaeological objects, as only open, porous structures can be re-treated and allow future consolidants to penetrate the artefact [34].

In this preliminary study, the drying behaviour and thermal and biological stability of selected biopolymers, as well as biopolymer-treated archaeological wood under different drying conditions, is presented to better understand the role of consolidant structuration in the long-term stability of waterlogged wooden artefacts. The effect of the wood environment on the drying behaviour is also examined. Originally, interest was confined to chitosan and cellulose nanocrystals in accordance with the past literature [34,52]; however, a recent publication by Janeček et al. [53] indicated an enhanced diffusion of negatively charged polymers within the wood cells; thus, alginate, a negatively charged polysaccharide, was also investigated.

## 2. Materials and Methods

### 2.1. Materials

Poly (ethylene glycol) (PEG, (M*_w_* = 400 g/mol), chitosan (M*_w_* = 60,000 g/mol), silicon wafers, agar, Müller–Hinton lysogeny broth, glycerol and acetic acid were all obtained from Sigma-Aldrich Chemical Company (St. Quentin Fallavier, France) and used as received. Alginate (M*_w_* = 100,000 g/mol) was donated by FMC Chemical International AG (Dublin, Ireland) and used as received. Cellulose nanocrystals (L = 100 nm, d = 5 nm) were kindly provided by CelluForce (Montréal, Canada). Both fresh and archaeological waterlogged oak wood (*Quercus robur*) used in this work was obtained from the Mary Rose Museum, for dimensions see electronic supplementary information (Appendix A). Non-pathogenic *Escherichia coli* (*E. coli*, DSM 5451-0599-001) and *Bacillus subtilis* (*B. subtilis*, DSM-1970-0110-001) were purchased from the German Collection of Microorganisms and Cell Cultures (DSMZ) GmbH (Braunschweig, Germany). Liquid nitrogen for cryogenic freezing was obtained from Kraiss & Friz e.K., (Remshalden, Germany). Water used in all experiments was obtained from an in-house water deionisation system. Preparation of films for initial microscopy studies and treatment of the wood samples is detailed in Appendix A.1 and Appendix A.2. Protocols for the biological tests are given in Appendix A.3.

### 2.2. Instrumentation

Standard light microscope images of polymer films were taken on a DM 750 Light Microscope from Leica Microsystems GmbH (Wetzlar, Germany). Determination of the freeze-drying ability of the biopolymer solutions was carried out on a freeze-drying microscope (FDM). The FDM comprised a transmitted light-polarising microscope (Axio Scope. A1, Carl Zeiss Microscopy Deutschland GmbH, Oberkochen, Germany) with a Lambda plate (polariser D, 90° pivotable, disengageable) and a freeze-drying stage (FDCS 196, Linkam Scientific instruments Ltd., Tadworth, UK), which can be cooled with liquid nitrogen and evacuated with a vacuum pump (Ilmvac GmbH, Ilmenau, Germany). The pressure was controlled using a Pirani gauge. A digital camera recorded images, which were analysed using Linksys 32 software (Linkam Scientific instruments Ltd., Tadworth, UK). Bulk freeze drying was carried out in an Alpha 2–4 LDPlus lyophiliser (Martin Christ, Osterode am Harz, Germany) for 2–3 h, where the pressure of the condenser was 0.01 mbar, and the temperature was −86 °C. Scanning electron microscope (SEM) images were obtained using a Vega 3 LM SEM (TESCAN, Brno, s.r.o., Czech Republic) at an accelerating voltage of 5 keV and a working distance of ca. 11 mm. Samples were coated with a gold layer 10–20 nm thick using a Q150R S sputter-coater (Quorum Technologies Ltd., Lewes, UK) prior to analysis. Thermal analysis of the treated samples was carried out on a Q200 thermogravimetric analyser from TA Instruments with a temperature ramp of 20–500 °C at 10 °C/min. Biological tests were carried out at 37 °C in either an Infors AG (Bottmingen, Switzerland) Type 225 agitator/incubator at 200 rpm for solution-based tests or a static incubator for plate-based tests for 18 h.

### 2.3. Freeze-Drying Protocol

To determine the suitability of the chosen biopolymers for freeze-drying applications, a drop of a 1 wt.% solution of each tested polymer was placed on a glass slide inside the chamber of a freeze-drying microscope. The samples were subjected to a temperature ramp from room temperature to −40 °C at 10 °C/min and held at this point for 8 min. The samples were then heated from −40 °C to −20 °C at 10 °C/min and then further from −20 °C to 0 °C at 1 °C/min with a pressure of 15 Pa where freeze-drying took place.

## 3. Results and Discussion

### 3.1. Freeze-Drying Behaviour of Chitosan, Alginate and CNCs

**Figure 1:**
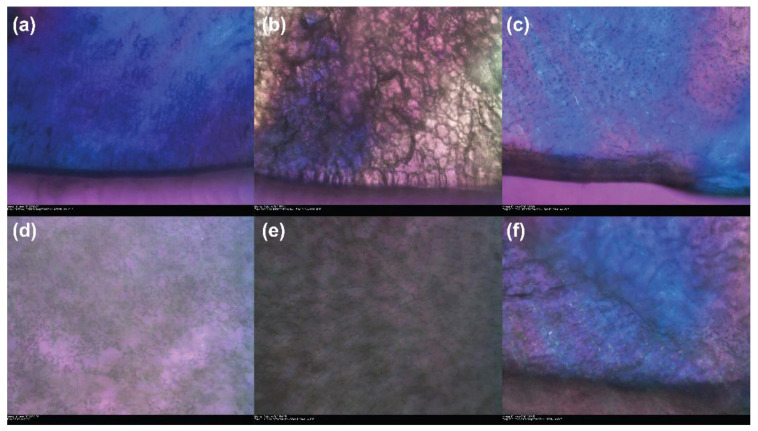
Images taken directly after freezing (**a**–**c**) and during freeze-drying (**d**–**f**) with a freeze-drying microscope of 1 wt.% solutions of chitosan (**a**,**d**), alginate (**b**,**e**) and cellulose nanocrystals (**c**,**f**), magnification 65×.

Controlled drying of waterlogged wood after treatment with a consolidant is often crucial to ensuring its prolonged dimensional stability. Thus, before examining the structuration of air-dried and freeze-dried polymers, it was necessary to optimise the freeze-drying conditions of each of the biopolymers and compare these with the state-of-the-art PEG consolidant. Previous experiments by Jensen and Schnell [54] and Wiesner and Giesler [39] have shown that PEG with M*_w_* ≤ 1500 g/mol does not freeze in standard commercial freeze-driers, as temperatures below −35 °C (the standard temperature cut-off) are required to ensure PEG remains frozen during drying. Between 1500 g/mol and 10,000 g/mol the solid temperature is in the region of −17 °C, making them suitable for freeze- drying applications. Low temperatures also extend the drying time, which is of particular importance for large objects. As mentioned previously, determining the freezing point of the polymer is important in preventing the consolidant from melting and losing any structuration before drying. The determination of the freezing point of each of the biopolymers found that at −19.4, −15.0 and −12.6 °C for chitosan, CNCs and alginate, respectively, structuration was observed throughout the sample, and this was taken to be the freezing point. It can, therefore, be seen that both alginate and CNCs fall into the same freezing point range as the 1500–10,000 g/mol PEG solutions, while chitosan is slightly lower, although well within the same range. The freezing and sublimation for all three biopolymers are shown in Figure 1.

**Figure A1:**
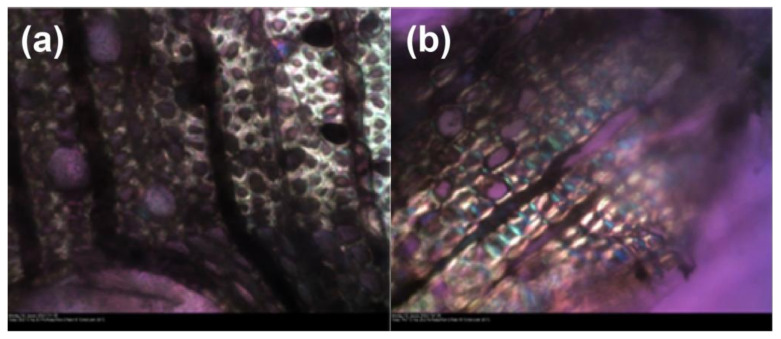
Optical microscope images of the wood with (**a**) 1 wt.% chitosan solution and (**b**) water, magnification 65×.

The freezing of alginate produces an open, porous structure characterised by thick alginate fibres and large pores (Figure 1a), which remains intact during the sublimation of water (Figure 1d). Heating the sample slowly during sublimation allowed the determination of the range over which freeze-drying was possible without melting the alginate. Evidence of sublimation was observed from −14.9 °C up to complete drying at −11.5 °C with no evidence of melting or collapse of the porous network structure. Chitosan (Figure 1b,e) displayed a more densely interlinked network structure with smaller fibres and pores (Figure 1b), which remains in the dried polymer (Figure 1e). Sublimation began at approx. −17.9 °C and all water was removed by −9.9 °C. However, some melting of the sample structure was observed around −12 °C (Appendix A Figure A1), indicating that the sublimation must take place below this temperature to avoid structural changes. Such ranges of drying behaviour without melting are important for the drying of larger objects, where maintaining the same, precise temperature throughout the object is more difficult.

The behaviour of the CNCs was slightly different to that observed for alginate and chitosan (Figure 1c,f). Upon freezing, CNCs form an ordered crystalline structure on the glass surface due to the strong H-bonding between them in solution (Figure 1c); this structure remains after the drying of the sample (Figure 1f). Interestingly, instead of displaying a drying front, as for alginate and chitosan, CNCs appear to instantaneously sublime after −15 °C, and a completely dry sample was observed at −14 °C. It is interesting to note that, as the perceived crystallinity of the biopolymers increases, the drying range of the samples decreases in the order chitosan > alginate > CNCs. This is potentially very useful for the future development of tailored consolidant systems, such as those described by Walsh et al. [26]. Greater control over the organisation and interactions of chains within the consolidant through chemical modifications giving rise to specific physical or covalent interactions between biopolymer chains could lead to better, more rapid freeze-drying and a greater chance of locking-in specific architectures within wood, leading to better stabilising mechanisms. Examination of the freeze-drying behaviour of the polymers has confirmed their suitability for the freeze-drying process and highlighted the unique structures formed by freezing of the polymers prior to drying. Considering many natural materials obtain their mechanical strength from the specific orientation of their component particulates and polymeric chains, such in situ structuration should influence the mechanical properties of the wood; an in-depth investigation on this is ongoing.

### 3.2. Comparison of Polymer Structure as a Function of Drying

#### 3.2.1. Drying Ex Situ

Initial optimisation of the freeze-drying conditions of 1 wt.% solutions of alginate, chitosan and CNCs showed that the freeze-drying conditions were similar to those previously reported for PEG. Large variation was, however, observed as a function of crystallinity. The freeze-drying microscope images also indicated a structuration of the consolidants during the drying process.

**Figure 2:**
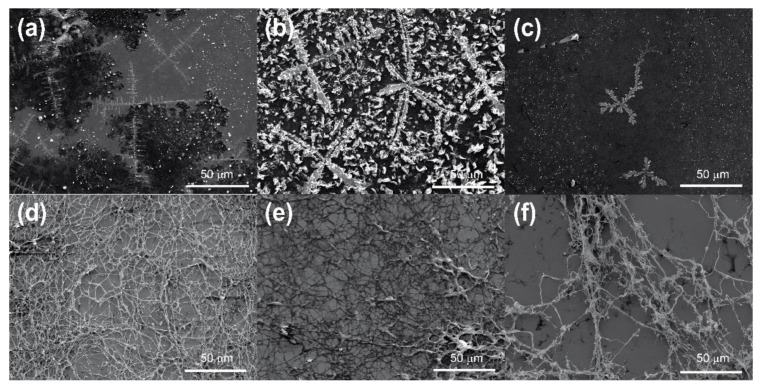
Scanning electron micrographs of 0.1 wt.% air-dried solutions of (**a**) chitosan, (**b**) alginate, (**c**) CNCs and 0.1 wt.% freeze-dried solutions of (**d**) chitosan, (**e**) alginate and (**f**) CNCs, magnification 1.38 k× (**a**–**c**), 890× (**d**–**f**).

While it is clear that structuration within the wood would be influenced by the structure of the wood cell and the chemical interactions between the wood and the consolidant, it is still important to examine the basic structuration of the polymer outside of the wood to better understand structuration of the consolidant as a general function of drying. In order to probe the effect of drying, samples of polymers were prepared on silicon wafers for analysis by scanning electron microscopy (SEM). In addition to freeze-dried samples of the selected solutions prepared en masse in a lyophiliser, in place of the microscope stage, air-dried samples were prepared to observe structural differences as a result of drying. The results of this experiment were analysed by SEM and are shown in Figure 2.

The SEM images illustrate a rather remarkable difference between the structure of air-dried (Figure 2a–c) and freeze-dried (Figure 2d–f) polymers. In each of the air-dried samples a thin film was formed, which on closer inspection had a random crystalline/amorphous appearance. The quantity of the crystalline domains appeared somewhat dependent on the crystalline content of the polymer, in that chitosan (being mainly amorphous) showed far fewer crystalline regions than alginate or CNCs, which have a higher degree of crystallinity. In examining the freeze-dried structures, it can be seen that all three polymers displayed an open network structure. This has been reported previously by Christensen et al. [34] for chitosan and cellulose nanowhiskers and was, therefore, to be expected. Such structuration of the polymers is extremely interesting. It is assumed that when dried in situ the air-dried polymers form wall coatings, while the freeze-dried polymers form network structures on or within the wood cells. From the point of view of wood conservation, network structures are the more desirable outcome. When wood cells are coated with polymer, but the polymer does not completely fill the lumen, there is the potential that cells collapse under mechanical stress as there is insufficient support from the consolidant. Network structures within the lumen have the advantage of being able to bridge the empty space, allowing the cells to better respond to and potentially offset mechanical stress, improving the mechanical stability of the structure, similarly to how mass transfer across the hierarchical structure of wood itself gives it its exquisite mechanical properties [55].

#### 3.2.2. Polysaccharide Treatment of Whole Wood Samples

**Figure 3:**
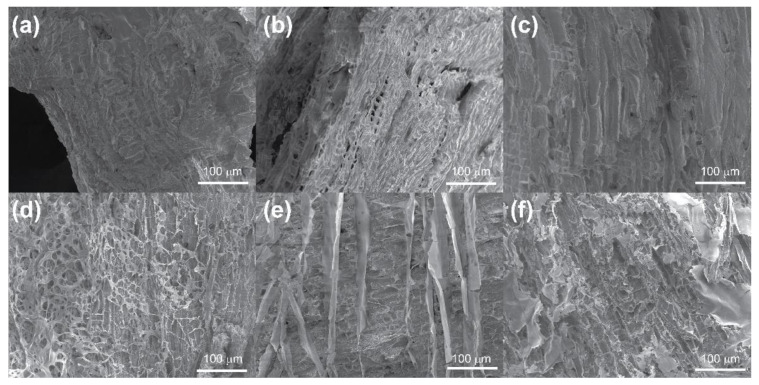
Scanning electron micrographs of archaeological wood with 0.5 wt.% air-dried solutions of (**a**) chitosan, (**b**) alginate, (**c**) CNCs and 0.5 wt.% freeze-dried solutions of (**d**) chitosan, (**e**) alginate and (**f**) CNCs, magnification 554×.

To examine this further, samples of archaeological wood of approx. 0.8 cm × 0.5 cm × 0.5 cm were treated with chitosan, alginate and CNCs over 14 weeks, dried and examined again by SEM, as seen in Figure 3.

As expected, the air-dried samples appeared to have no polymer coating. However, visual observation of a shiny coating on the surface of the wood confirmed the presence of the biopolymer coating. On the other hand, freeze-dried samples dried were visibly coated with the open, porous network observed in Figure 3d–f. In the case of chitosan and CNCs this structure appeared random, although similarities between the network and structure of the wood cells could be seen and seemed to form independently of the cell wall. In the case of alginate, however, the structuration closely followed the natural pattern of the wood, indicating a polymer–wood adhesion effect. Such behaviour poses the question of whether alginate is more adhesive to the wood structure than chitosan or CNCs as a function of surface chemistry or charge, as mentioned previously by Janeček et al. [53].

#### 3.2.3. Drying Behaviour in the Wood Cells

**Figure 4:**
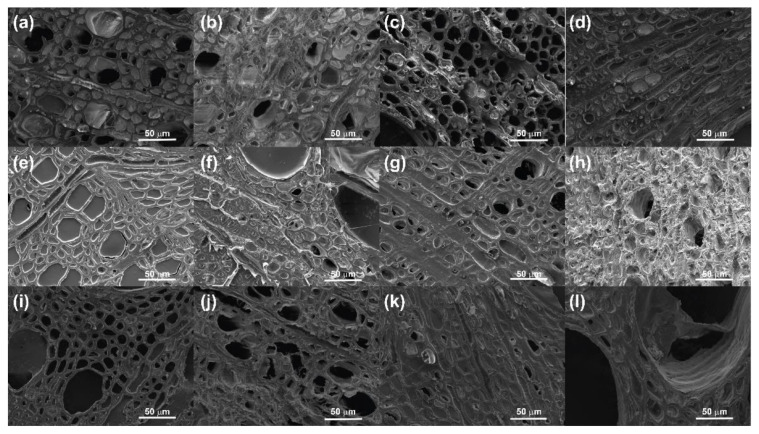
Scanning electron micrographs of chitosan-treated fresh wood after air- (**a**) and freeze-drying (**b**) and archaeological wood after air- (**c**) and freeze-drying (**d**); alginate-treated fresh wood after air- (**e**) and freeze-drying (**f**) and archaeological wood after air- (**g**) and freeze-drying (**h**); and CNC-treated fresh wood after air- (**i**) and freeze-drying (**j**) and archaeological wood after air- (**k**) and freeze-drying (**l**), magnification 1.11 k×.

Large-scale treatment poses certain issues related to molar mass and diffusion within the wood and makes interpretation of the in situ behaviour more complex. To simplify these observations, thin layers (ca. 10 μm) of both fresh and archaeological wood were cut using a microtome. Fresh wood was used in this case as a control to better understand polymer interactions with the cell walls. These pieces were then immersed in distilled water for approx. 10 months, ensuring complete waterlogging. The wood was removed from distilled water, immersed in 0.5 wt.% solutions (to reduce sample viscosity) of CNCs, chitosan or alginate for 30 h and, finally, subjected to air-drying or bulk freeze-drying. The results of SEM investigation of both the archaeological and fresh wood are shown in Figure 4.

While Figure 3 suggests it can be difficult to verify the presence of the air-dried polymers within the wood cells, by cutting the samples into thin sections before treatment, so that the cells, lumens and pits are visible, the mode of attachment of the polymers to the cells was more evident. Figure 4a–d show sections of chitosan-treated fresh (Figure 4a,b) and archaeological (Figure 4c,d) wood after air- and freeze-drying. Both air- and freeze-dried samples (Figure 4a–d, respectively) were markedly different. Chitosan-treated air-dried fresh oak appeared to leave no visible coating and little filling of cells and lumens, although it is likely from visual observation that a thin film covered the wood, while the archaeological oak showed coverage by a thick network, which could be seen protruding from the cells. These protrusions may have been damage due to the vacuum of the SEM, suggesting that the films may not be very mechanically stable. In the case of the freeze-dried fresh oak, there was significant structured polymer observed both with the cells and lumen and along the secondary cell wall structure. A similar pattern was observed with the archaeological oak, although to a lesser extent, indicating the possibility that cell wall components to which the chitosan will attach are reduced during waterlogging or degradation. Another potential explanation is that the chemical structure of chitosan is heavily dependent on the environment, which may interrupt the formation of network structures, instead allowing only film-like coatings that are difficult to observe in the SEM.

The alginate-treated wood (Figure 4e–h) showed even better uptake of the polysaccharide at the same concentration and over the same treatment duration. Both fresh (Figure 4e) and archaeological (Figure 4g) air-dried samples showed significant film formation in the cells, lumen and pits. The number of filled cells in the archaeological oak was slightly reduced compared to the fresh wood, which may have been due to natural wood variation or the leaching of extractives to which alginate may bind, thus reducing its affinity for the cell wall. The freeze-dried samples of fresh (Figure 4f) and archaeological (Figure 4h) showed a similar pattern of filling. The fresh oak showed a mixture of film formation and structuration of the alginate both within the cells and along the cell wall. The structuration of the alginate in the environment of the archaeological oak was much more pronounced. Figure 4h showed that almost every cell was filled with a randomly structured network that also followed the cell wall structure above the wood. From this specific behaviour, it can be assumed that alginate is not only a suitable consolidant due to its ability to form a network structure within the wood cells but also because it creates structure where there is no template. This suggests it may also be suitable for gap-filling in the wood morphology with a network that strongly mimics that of the “missing” wood.

Lastly, Figure 4i–l show images of wood treated with CNCs. As with the air-dried samples of chitosan and alginate, film formation could be observed in both the fresh (Figure 4i) and archaeological (Figure 4k) samples. As seen in chitosan-treated samples, the film formation seemed more extensive in the archaeological rather than the fresh sample; however, as before, this might have been due to natural variation or the affinity of the CNCs for the cell wall after removal of extractives. The freeze-dried samples showed a similar tendency, with lots of structured networks visible along the cell wall and within some cell lumen in the fresh oak, although less than observed for chitosan, (Figure 4j), and some film formation and structuration was visible in the archaeological oak (Figure 4l), although, again, to a reduced degree. The treatment of the wood with the biopolymers certainly produced some interesting results and avenues for further study. It would appear from the SEM images that the alginate fills the wood cells better within the treatment window compared to the solutions of chitosan and CNCs. This is completely at odds with their molar masses and the dimensions of the CNCs, as well as the apparent viscosity of the solutions, as given in Table 1.

**Figure A2:**
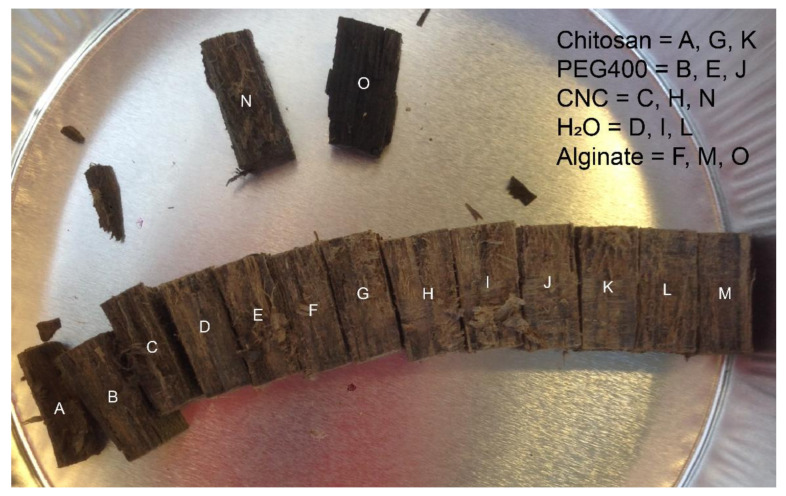
Visual observation of samples of archaeological wood before treatment.

**Figure A3:**
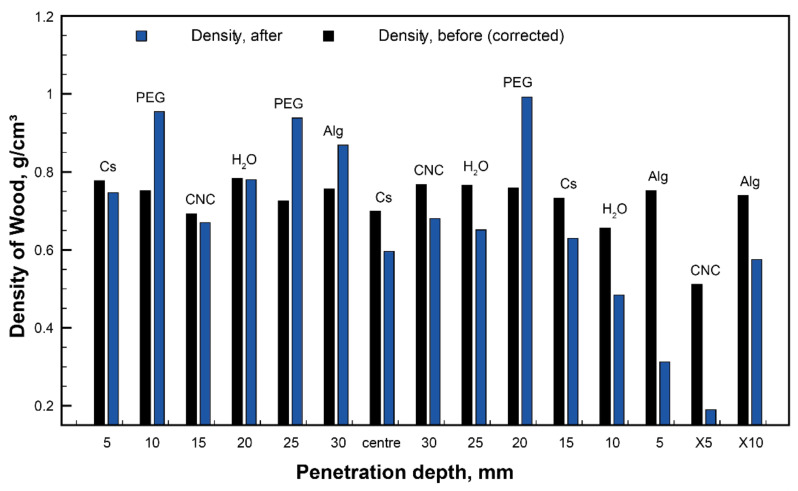
A histogram of the density of the wood samples before treatment, after soaking and after drying as a function of penetration depth and polymer solution, showing the effect of the drying conditions on the density.

Several works have suggested that the dimensions of the CNCs (5 nm diameter × 100 nm length) should allow them to pass easily into the cells of the wood; however, there is also evidence of a filtering effect of the CNCs by the wood, meaning penetration only occurs very close to the surface of the wood [56]. Christensen et al. have shown that chitosan solutions with M*_w_* of 6000–60,000 g/mol can penetrate archaeological wood, although the diffusion is significantly improved as the M*_w_* decreases [36]. Despite diffusion into cells, given their molar masses and dimensions with respect to CNCs, it may be difficult for these biopolymers to penetrate the cell wall. Archaeological wood has an average cell wall pore size of 10–20 nm [43], which may explain the filtering effect of the CNCs and may also prevent large polysaccharides from penetrating the cell wall in the same manner as PEG. Thus, further examination of the size vs. structure relationship of these polysaccharides is warranted to enhance the likelihood of cell wall penetration without compromising structural integrity. In general, the results obtained here confirm that CNC and chitosan do diffuse into and coat the wood cells, forming films and network structures to a greater or lesser degree, and density measurements of the wood after treatment and freeze-drying (Figure A2 and Figure A3) suggest there is a filtering effect at play. When wood from the outer edges of an archaeological wood segment was treated with chitosan and CNCs (up to 15 mm depth) the density of the wood before and after drying was very similar, suggesting some infiltration of the polymer into the wood structure. However, samples deeper into the wood (greater than 30 mm) showed that the density of the wood after drying was lower than the untreated wood, indicating shrinkage and improper penetration. These results are similar to results obtained for pure water and are in direct contrast to the data obtained from the PEG400 control, which showed a bulking effect even in samples obtained from 50 mm into the wood segment, which would be expected to be more dense and more difficult to penetrate.

Alginate, with a molar mass of 100,000 g/mol, showed a much greater affinity for the wood surface chemistry than either CNCs or chitosan, resulting in a greater degree of coverage of the wood after treatment. By cutting thin samples of the wood, the viscosity contribution was reduced as the treatment does not need to penetrate the entire wood structure, just the open cellular structure. Despite this, it would still be expected that a lower-viscosity solution such as CNCs (1.92 mm^2^/s) or chitosan (12.62 mm^2^/s) would have less difficulty penetrating all aspects of the wood structure than alginate (68.68 mm^2^/s). In addition to their viscosities, these three solutions have significantly different charges, with chitosan being positively charged, CNCs slightly negatively charged, and alginate strongly negatively charged. Janeček et al., noted that surface charge, more than viscosity, affected the diffusion of a variety of polymers aimed at reinforcing lower-grade woods [53]. In this work, negatively charged polymer solutions appeared to diffuse better into the wood than positively charged or zwitterionic polymers. It would seem that the same is true in this case. Again referring to the density measurements shown in the Appendix A, Figure A2 and Figure A3, samples obtained from a depth of 30 mm into the wood showed a higher density after treatment with alginate, similar to the data obtained from PEG400; however this effect was no longer visible in samples obtained from more than 50 mm into the wood (less than 30 cm from the inner protected side of the wood, Figure A3), which suggests that charge assists to a certain point but is eventually counteracted by polymer size. With respect to why negatively charged polymers diffuse to a greater extent in wood, it may simply be the case that the negatively charged polymers, containing carboxyl groups, are undergoing esterification reactions with free OH groups on the wood surface under the acidic conditions present within the wood. This would lead to covalent interactions with the wood in comparison to CNCs and chitosan, which interact through physical interactions. Such variation in the different properties of the polymers in the wood cells as a function of drying and of the type of wood used begs the questions of how dependent structure formation is on the environment and how the films and networks interact with the components of the wood cell wall.

### 3.3. Thermal Stability of Fresh and Archaeological Woods as a Function of Treatment

In order to understand the structuration of the biopolymers within the wood cells, some idea of their interactions with the wood surface was required. To gain this information, thermogravimetric analysis (TGA) was employed. Several publications have highlighted the utility of thermal analysis in understanding the effect of treatment on the natural behaviour of the wood [57,58,59]. In these works, increases in the maximum pyrolysis temperature (MPT) of the wood were linked to a restorative effect of the treatment, enhancing the ability of the wood to resist thermal degradation. Decreases in MPT were linked to incompatibility and a negative impact of the treatment on the wood. By observing which of the characteristic wood peaks was altered, an understanding of the affinity of the treatments for various cell components could also be obtained.

**Figure 5:**
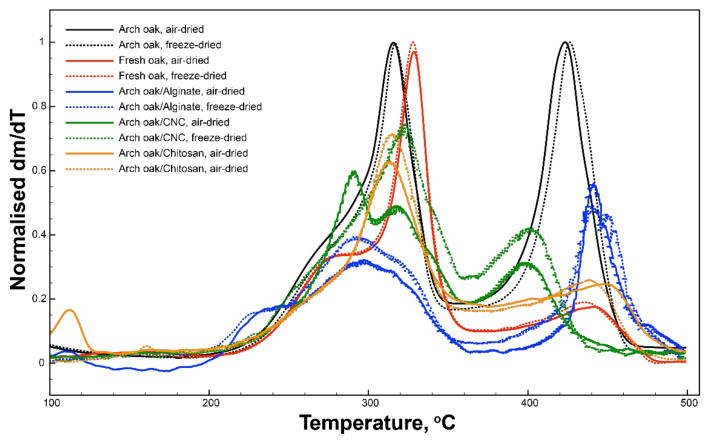
Differential thermograms (DTG) of fresh (red) and archaeological (black) wood before treatment, alginate-treated archaeological wood (blue), CNC-treated archaeological wood (green) and chitosan-treated archaeological wood (orange) after air- and freeze-drying.

Figure 5 shows the differential thermograms (DTGs) of fresh and archaeological oak before and after treatment with 0.5 wt.% solutions of chitosan, alginate and CNCs. In Figure 5, there are three principle characteristic peaks in the DTGs of all four control samples, both air- and freeze-dried fresh and archaeological oak: a peak around 275 °C for the amorphous cellulose and hemicellulose content, which is more pronounced in the fresh wood due to lack of degradation, a peak at 315–330 °C, representing crystalline cellulose and a peak at 405–445 °C, representing lignin. The degree of shifting of the peaks is an indication of the crystallinity or the state of degradation of the component. The negative shift of the lignin peak between fresh and archaeological samples before treatment confirms the observation by Colombini et al. that lignin does not remain unchanged throughout the degradation process [60].

In the chitosan-treated samples, there was no significant shift of the cellulosic peaks after treatment; however, there was a marked difference in the lignin peaks, which shifted from 405 to 450 °C in the air-dried sample and 410 to 440 °C in the freeze-dried sample. The shifting of the lignin peak to higher temperatures after treatment suggests interactions of the chitosan with the lignin, improving its thermal properties to pre-degradation values. However, no significant interaction of the chitosan with the cellulosic components was observed. Due to the decreased cellulosic content of the degraded wood, affinity for the lignin skeleton is an important attribute in a consolidant for very degraded wood.

In direct contrast to chitosan-treated samples, CNC-treated samples showed no shift in the lignin peak, and all changes were observed at the cellulosic peaks. In the freeze-dried sample a broadening of the crystalline cellulose peak was observed, while the air-dried sample showed a change in the amorphous peak. It would, therefore, appear that the structuration of the cellulose consolidant has a strong effect on its thermal stability. This correlates with the organisation of the cellulose, or lack thereof, observed in the SEM. Due to its chemical structure, CNCs appear to have a greater affinity for the cellulosic component than lignin, which is to be expected. It is also interesting to note that their organisation on the wood surface seems to play a role in the thermal stability of CNC-treated wood.

Finally, the DTG plots of alginate-treated wood showed that changes in both the lignin and cellulose peaks occurred in these samples. It seems that the extensive wall coating of the more amorphous alginate on the cellulose of the archaeological wood results in a noticeable negative shift in the thermal degradation peak of the crystalline cellulose towards the amorphous cellulose region. This indicates that the addition of alginate to the wood structure reduced the thermal stability of the cellulose. On the contrary, alginate induced a positive shift in the lignin peak from 409 to 442 °C in both the air- and freeze-dried samples. As observed with the chitosan-treated wood, after treatment, the alginate returned the lignin peak to a similar value as lignin in fresh wood.

From these results it appears that chitosan interacts solely with the lignin component, and CNCs solely interact with the cellulosic material, while alginate appears to interact with both of the major components. Although alginate appears to reduce the thermal stability of the cellulosic matrix, the reduced prevalence of celluloses in degraded wood mean that this may not always be an issue of high concern in consolidant selection. The results of this thermal study highlight the potential unsuitability of CNCs as an archaeological wood consolidant if the main point of attachment is through cellulose. This issue is visually evident in Figure 4i–l, where the surface coating on the CNC-treated fresh wood samples, as seen in Figure 4i,j, appear much greater than those in Figure 4k,l, which represent the treated archaeological wood.

### 3.4. Effect of the Biological Activity of the Polymer

Initially, the biological activity of chitosan, alginate, CNCs and PEG400 was observed in the presence of untreated archaeological wood over 4 weeks, during a treatment protocol. Although this does not provide quantitative data of anti-microbial activity, it is an indication of the suitability of the polymer for the treatment process without the need to add anti-microbial agents. The results of these observations are shown in Appendix A, Table A1.

While PEG400 has no native anti-bacterial properties, little biological activity was observed in the PEG400 solutions over the course of the treatment. This is likely due to the observations reported by Björdal and Nielsen that, above a certain concentration, PEG acts as an oxygen barrier, preventing the growth of aerobic bacteria, thus appearing to limit bacterial growth [61]. In reality, it simply requires more time for the oxygen to penetrate the PEG and allow bacteria and fungi to grow; once exposed they proliferate quite rapidly, forming a biofilm.

**Figure 6:**
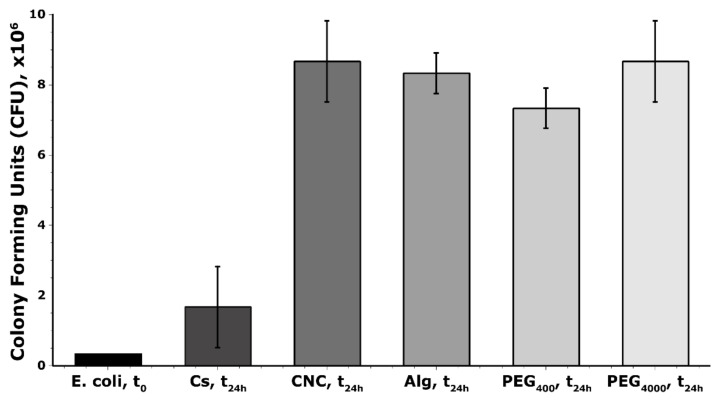
Bar chart showing the number of *E. coli* after 24 h incubation with chitosan, CNCs, alginate, PEG400 and PEG4000.

For a more quantitative idea of the anti-bacterial properties of the polymers chosen for this study, biological tests were performed as described by Walsh et al. on *E. coli* in lysogeny broth for 24 h at 37 °C [26]. Here, 50 wt.% PEG4000 was also tested in addition to the previously chosen polymers to understand if there was a marked difference in anti-bacterial efficiency as a function of chain length. The results of these tests are shown in Figure 6.

As expected from Figure 6 it is clear that, regardless of the observations made in the treatment bath, chitosan is the best treatment option in terms of quantifiable anti-bacterial efficiency. CNCs and alginate do not show significant anti-bacterial properties. Interestingly, PEG4000 and CNCs both offer similarly low resistance to colony formation, followed by alginate and PEG400. Chitosan offered the greatest resistance to bacterial colony formation.

**Figure A4:**
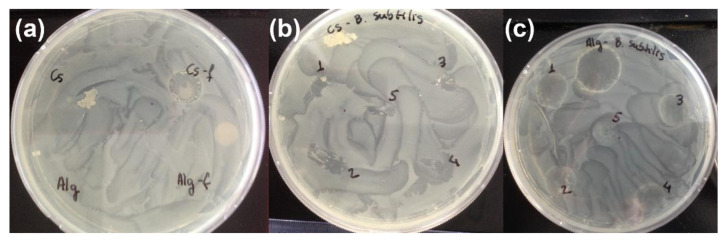
Photographs of biological resistance tests of (**a**) freeze-dried and air-dried chitosan and alginate against *E. coli*, (**b**) freeze-dried chitosan and (**c**) freeze-dried alginate against *B. subtilis*.

A major question in relation to this work is whether the structure of the polymer formed during drying affects its behaviour as a consolidant material, more specifically if structured polymers are more effective than those without a defined structure. Examples from the natural world, such as duck feathers, cicada wings and nacre, show that the structure of biomaterials often leads to enhanced water repellency, bactericidal properties or mechanical properties, respectively [62,63,64]. To test whether the chosen polymers can display differences in microbial resistance as a function of organisation, an example of a good and a poor anti-bacterial consolidant were subjected to freeze- and air-drying and then to further testing against *E. coli* and *B. subtilis*. As it was evident that chitosan and alginate possess more desirable consolidant qualities than CNCs, as validated by a similar realisation in a recent paper by Antonelli et al. [56], who noted a filtering effect of CNCs when used for archaeological wood conservation, only chitosan and alginate were examined for the rest of the study. Agar plates were prepared, to which cultured *E. coli* was added. An air-dried film and a freeze-dried foam of both chitosan and alginate were placed on each plate and allowed to incubate overnight at 37 °C. This experiment was repeated in triplicate, and an example of the results shown in Figure A4 of Appendix A.

Figure A4a shows that there was no change in the anti-bacterial behaviour of these polymers as a function of their structure. The freeze-dried polysaccharides were also tested against *B. subtilis* (Figure A4b,c) with similar results. Regardless of organisation, chitosan remains strongly anti-bacterial in the contact region, while alginate is quickly dissolved by the bacterial medium and colonised by the bacteria. This illustrates that, at least in the case of these biopolymers, there is no clear enhancement of bacterial resistance as a function of structure.

### 3.5. Sensitivity of the Structuration to Environmental Influences

A principal question arising from this study, why alginate attaches better to the wood samples than the other biopolymers tested, remains to be answered. Does the enhanced adhesion relate to surface charge or chemistry? Can this supersede viscosity and molar mass in the treatment of wooden objects? Or is the behaviour of the alginate more susceptible to the environment inside the wood, the presence of salts or extractives, which act to enhance the structuration of the alginate polymer? Or is there a combination of different effects?

**Figure 7:**
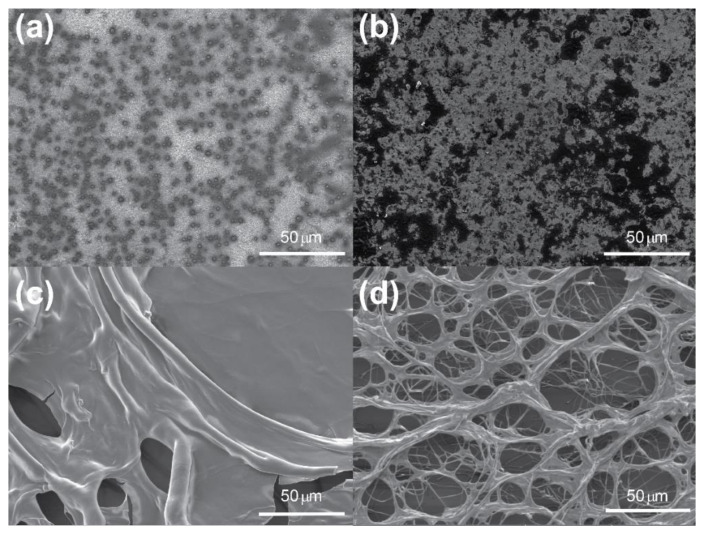
Scanning electron micrographs of air-dried chitosan (**a**) and alginate (**b**) and freeze-dried chitosan (**c**) and alginate (**d**) in the presence of wood extractives, magnification 1.38 k×.

In order to examine the susceptibility of the consolidants to their environment and the effect on their eventual structure, pieces of archaeological oak approximately 0.5 cm × 0.5 cm × 0.5 cm were added to 1 wt.% solutions of chitosan or alginate and allowed to soak for approximately 14 weeks. After this time the wood was removed, and the solution was diluted 10-fold. A drop of this solution was placed onto freshly cleaned silicon wafers and dried by either air- or freeze-drying; the resulting structures were imaged by SEM and are shown in Figure 7.

As with Figure 2a–c, there was no structuration of the air-dried samples, although the crystalline content appeared to be greater. This is likely a result of an increased concentration of salts and sugars present in solution from the wood. The difference in structure in the freeze-dried samples is much more evident. Despite applying a freeze-drying procedure as before, there was no defined structure in the chitosan sample (Figure 7c). The presence of salts and extractives from the archaeological wood appeared to contribute to the loss of the discreet network structure observed in Figure 2d, with chitosan now forming a thick film with some larger pores. Alginate, on the other hand, appeared to retain and even improve its network structure as a function of the environment with evidence of fibrillation of chains, as seen in Figure 7d, that cannot be observed in Figure 2e. Such structural motifs are similar to those displayed by proteins such as collagen or fibrin, whose structures are linked to their exquisite mechanical properties. The environment of the wood is, however, extremely complex, and further investigation of the interaction of the biopolymers with individual components of the wood will be necessary to properly understand the role of the environment in their behaviour. As a preliminary result, however, such an enhancement in the structuration of alginate is extremely interesting and points to the need for more detailed investigation of alginate as a consolidant material or a base for the design of more responsive consolidants. Given that alginate has long been used in bone repair, another complex environment, with much success, the positive properties of alginate applied to the conservation of wooden materials are not entirely unexpected.

## 4. Conclusions

In this work the biological, morphological and thermal properties of several biopolymer-treated wood samples have been investigated for the development of new and efficient consolidants for waterlogged wood. The morphology of the treatments upon drying, how this is affected by the drying protocol and whether this plays a role in the biological resistance or thermal stability of the treated wood was also investigated. It is evident from this work that the drying protocol, and the resulting structure, do not play a key role in the biological resistance of the biopolymer consolidants tested here but may influence the thermal stability of the treated wood. After freeze-drying in the presence of wood extractives, alginate formed a more fibrillated network structure, which may aid the overall dimensional stability of the treated wood. This enhanced structure was absent in the other polymers tested, as well as the air-dried samples, suggesting alginate is more resistant to the effect of sugars and other polymers present in the wood. Both alginate and chitosan appeared to enhance the thermal stability of the lignin component of the treated wood, an important factor in choosing consolidants for archaeological wood where the cellulosic component may be significantly reduced. Thus, the results obtained here suggest that alginate, along with chitosan, are suitable polymers for further development of consolidants for archaeological wood, particularly with respect to freeze-drying. However, a better understanding of the molar mass–3D structure relationship of these polymers must be obtained, alongside development of modification strategies to enhance the microbial resistance of alginate, before systematic studies in archaeological wood can be undertaken. Thus, alginate and chitosan-based consolidants should be, or should continue to be, the focus for future sustainable consolidant development.

## Figures and Tables

**Table 1:** Measured viscosity of the polymers used in this work, all measurements recorded at 25 °C.

Polymer	Concentration (wt.%)	Viscosity (mm^2^/s)
PEG (400 g/mol)	50	11.27
Sodium alginate (100,000 g/mol)	1	68.68
Chitosan (60,000 g/mol)	1	12.62
CNCs (5 nm × 100 nm)	1	1.95

## Data Availability

Supporting data will be made available here: Z.W.-K., I.S., J.d.S.G., G.E. and L.A. (6 December 2021). Morphological study of bio-based polymers in the consolidation of waterlogged wooden objects. Retrieved from osf.io/8qae4 (accessed on 6 December 2021).

## Appendix A

### Appendix A.2. Preparation of Polymer Films for Scanning Electron and Optical Microscopy

Optical microscopy samples were drop-cast on mica surfaces, which were freshly delaminated before deposition. Scanning electron microscopy samples were also drop-cast but on silicon wafers, which were cleaned with piranha solution and then stored in ethanol prior to use. In all cases the biopolymer solutions and PEG were diluted 1000-fold before casting in order to be able to see discrete structures on the sample surface.

### Appendix A.3. Treatment of the Wood Samples with Biopolymer Solutions and PEG

Biopolymer solutions were prepared at a concentration of 0.5 wt.% while PEG400 was prepared at 50 wt.% in deionised water. A block approximately 0.8 cm thick was cut from a piece of waterlogged untreated archaeological wood and then further divided into cubes of approximately 0.8 × 0.5 × 0.5 cm^3^. Each cube was labelled and then placed in a polymer solution and stored in the refrigerator for 14 weeks before drying and weighing. The samples before treatment can be seen in Figure A2.

The dimensions of the samples were measured before and after drying (by lyophilisation) to understand density changes as a function of treatment. Samples were taken from a slice of *Mary Rose* oak beam and treated with various polymers at different penetration depths, meaning the distance from the outer surface. The order of the samples corresponds to the order seen in Figure A2, where X5 and X10 were the last 5 mm (N, Figure A2) and last 5–10 mm (O, Figure A2) of a second slice to have the same number of samples for each treatment. These would be of equivalent degradation to L and M, as seen in Figure A2. Penetration of bio-based consolidants was compared to that of pure water and PEG400. The depths in Figure A3 are given as the distance from the outer edge; the samples up to the centre were obtained from the exposed side of the wood, and the samples after the centre were from the inner protected side of the beam; this was estimated to be the denser side of the wood as it was less obviously degraded.

### Appendix A.4. Visual Observation of Anti-Bacterial Properties of Treatment Solutions

**Table A1:** Table of the biological activity observed in the samples during treatment relative to the treatment solutions used; numbering is related to samples seen in Figure A2.

Samples	Treatment	Presence and Degree of Biological Activity
1–3	H_2_O	(d) Small bacterial filaments (i) Mild biological activity (l) Moderate biological activity
4–6	50 wt.% PEG400	(b) Does not sink, no biological activity (e) Does not sink, no biological activity (j) Sinks, no biological activity
7–9	0.5 wt.% chitosan	(a) No biological activity (g) No biological activity (k) No biological activity
10–12	0.5 wt.% CNCs	(c) Moderate biological activity (h) Moderate biological activity (n) High biological activity
13–15	0.5 wt.% alginate	(f) Low biological activity (m) Mild biological activity (o) Mild biological activity

### Appendix A.5. Protocols for Biological Testing

Biological activity within the polymer solutions was tested according to the following procedure adapted from Tan et al. [65]. A total of 1 mL of the bacterial preculture (*E. coli* or *B. subtilis*) was added to 10 mL of Luria–Bertani lysogeny broth in a 50 mL Erlenmeyer flask and incubated overnight at 37 °C while being agitated continuously at 200 rpm. After incubation, a solution containing 10^7^ CFU/mL of microorganism (where optical density at 600 nm (OD_600 nm_) = 0.01 to *E. coli*) was prepared. Into each well of a multiwell plate (tissue culture plate, 24 well) 200 µL of the bacterial suspension containing 10^7^ cells/mL, 1 mL of fresh LB and 1 mL of the polymer solutions (0.5 wt.% for the biopolymer or 10 wt.% for PEG) were added. In order to minimise contamination of the samples 6 individual samples with three replicates were prepared for every polymer solution so that repeated sampling of the solutions would not be necessary to monitor bacterial growth over time. Once prepared the initial OD_600 nm_ of the solutions was measured and the 1st row of solutions discarded. The multiwell plate was then returned to the incubator and agitated at 200 rpm and 37 °C. The plates were removed and the OD_600 nm_ measured in triplicate every 2 h until 8 h, and then samples were taken again at 24 h. The inhibitory index of the samples can then be calculated from the following Equation:

$$Inhibitoryindex\left(\%\right)=[1-\left(\frac{A_{sample600\;\mathrm{nm}}-A_{sample0600\;\mathrm{nm}}}{A_{reference600\;\mathrm{nm}}-A_{reference0600\;\mathrm{nm}}}\right)]\times 100$$

where $A_{sample600\;\mathrm{nm}}$ is the absorbance of bacterial medium with sample after incubation, $A_{sample0600\;\mathrm{nm}}$ is the absorbance of bacterial medium with sample before incubation, $A_{reference600\;\mathrm{nm}}$ is the absorbance of bacterial medium after incubation, and $A_{reference0600\;\mathrm{nm}}$ is the absorbance of bacterial medium before incubation.

### Appendix A.6. Testing the Effect of the Biological Activity of the Polymer

Agar plates were prepared, to which cultured *E. coli* was added. On each plate an air-dried film and a freeze-dried foam of both chitosan and alginate were placed and allowed to incubate overnight at 37 °C in the static incubator. This experiment was repeated in triplicate, and an example of the results shown in Figure A4.
